# Management of patients requiring high-flow nasal cannula in a pediatric observation unit: a single-center experience

**DOI:** 10.1186/s12887-026-06525-y

**Published:** 2026-01-26

**Authors:** Evelyn Zhang, Yvette Calderon, Czer Anthoney Lim, Diana Hou Yan

**Affiliations:** 1 Children’s National Hospital, Washington, DC USA; 2 Department of Emergency Medicine, Thomas Jefferson University, Philadelphia, PA USA; 3 Department of Emergency Medicine, Columbia University Vagelos College of Physicians and Surgeons, New York, NY USA; 4 Department of Emergency Medicine, Ichan School of Medicine at Mount Sinai, New York, NY USA; 5 Icahn School of Medicine at Mount Sinai, One Gustave L Levy Place, Floor 12, New York, NY 10029 USA

**Keywords:** High-flow nasal cannula, Pediatric observation unit, Length of stay, Respiratory surge

## Abstract

**Background:**

Due to widespread shortages of pediatric intensive care unit (PICU) beds during the 2022–2023 respiratory virus surge, a protocol was implemented to identify and manage patients on high-flow nasal cannula (HFNC) in a pediatric observation unit (POU). This study aims to describe the characteristics and outcomes of patients who received HFNC in the observation unit.

**Methods:**

We performed a retrospective chart review of all patients admitted to a community pediatric observation unit who received HFNC between October 2022 and January 2023. Patients were grouped by disposition from the observation unit, either discharged home or transferred to PICU. Descriptive analysis was conducted, presented as median and interquartile range (IQR) or number and percentage.

**Results:**

Among 18 patients, 6 patients were discharged from the POU and 12 were transferred to the PICU, with an escalation rate of 66.6%. The most common pathogens in the sample were RSV (10 [55.5%]) and influenza (5 [27.8%]). Final diagnoses included bronchiolitis (8 [44.4%]), pneumonia (6 [33.3%]), bronchiolitis with secondary pneumonia (3 [16.7%]), and asthma exacerbation (1 [5.6%]). Median length of stay in the observation unit was 33.9 h (IQR 16.5–48.8). 4 of the 12 patients transferred to the PICU required higher forms of respiratory support, but none required intubation. There were no deaths in our sample, and no patients returned to the emergency department within 7 days of discharge. 293.2 PICU-bed-hours were saved.

**Conclusions:**

In this study, several patients were able to be de-escalated from HFNC in a community pediatric observation unit setting without severe complications, including a zero-intubation rate. In a resource-strained setting, managing patients on HFNC in an observation unit may be a reasonable alternative to conserve PICU resources.

## Background

Respiratory disease is a major cause of morbidity and mortality in children worldwide [1], as well as a consistently large cost burden on the US healthcare system [2]. High-flow nasal cannula (HFNC) is a form of respiratory support that has increased utilization in pediatric patients. It is safe and effective in children [3–5] and is an option between low-flow oxygen therapy and continuous positive airway pressure (CPAP) or bilevel positive airway pressure (BiPAP) [3]. Many studies have shown the benefit of HFNC in children with bronchiolitis, improving respiratory rate, fraction of inspired oxygen (FiO_2_), and oxygen saturation [6], as well as breathing pattern and work of breathing [6–8]. Other common causes of respiratory distress among children, such as asthma exacerbation, can also benefit from HFNC therapy [9, 10].

Traditionally, HFNC is used in the pediatric intensive care unit (PICU), but it has been effectively used in other hospital settings as well, including on non-tertiary inpatient units [4, 7, 11]. However, the triple epidemic of respiratory syncytial virus (RSV), influenza, and COVID-19 in children during the 2022–2023 respiratory viral season resulted in widespread shortages of pediatric hospital beds, with up to 75% of pediatric beds occupied [12], leading to increased wait times and boarding in emergency departments across the United States [13]. In response to this bed shortage, our hospital developed guidelines for managing HFNC patients in a pediatric observation unit (POU). Observation units are hospital areas dedicated to ongoing evaluation and management of patients, usually those with minor illnesses that need care for a well-defined, brief period of time that traditionally would have required inpatient admission [14]. Conditions ranging from asthma to dehydration to psychiatric conditions have been successfully managed in an observation unit setting [15, 16], with some studies demonstrating decreased hospital admissions and length of stay from managing pediatric respiratory conditions in a POU [17, 18]. No studies to date have described HFNC use in a POU.

Expanding HFNC use to community healthcare centers such as a POU would allow more patients to receive appropriate treatment in a timely manner and save PICU beds for those with more serious illnesses. This would not only be beneficial for future respiratory surges, but also has the potential to improve resource management during respiratory virus season annually. The aim of this study is to describe the characteristics and outcomes of patients who received HFNC therapy in a POU during a respiratory surge that limited ICU resources.

## Methods

### Study design and setting

We performed a single-institution retrospective chart review at a pediatric observation unit (POU) that is attached to a community-based pediatric emergency department (PED). The POU consists of 5 beds within a space adjacent to the 7-bed PED. During the day, the POU is managed by an in-house pediatric hospitalist with pediatric emergency medicine attendings available for consult. Overnight, a pediatric emergency medicine attending covers both the PED and the POU. One registered nurse is assigned exclusively to the POU at all times. A respiratory therapy team provides coverage for the entire hospital, including the POU. There are no pediatric medical inpatient or critical care units on site, with the nearest PICU at least 30 min away. The POU has an average annual volume of approximately 700 admissions, while the PED manages about 13,000 visits per year.

**Fig. 1:**
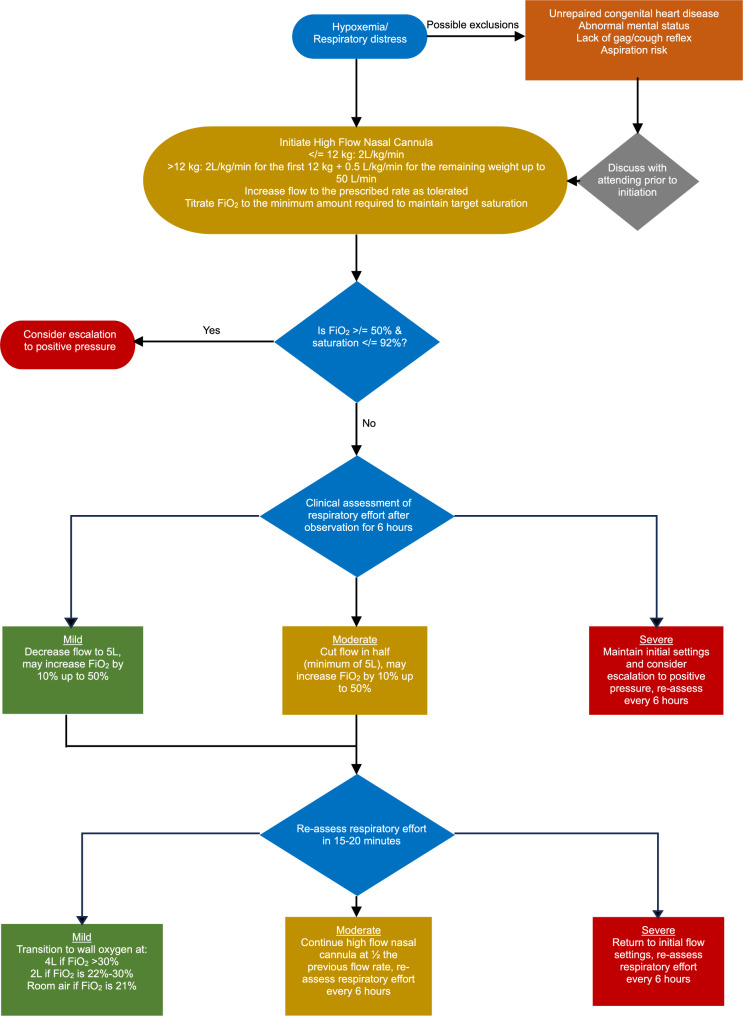
HFNC treatment protocol in the pediatric observation unit

Due to limited pediatric inpatient bed availability during the 2022–2023 respiratory virus surge, a HFNC treatment protocol was adapted from Betters et al. [19] and modified specifically for the POU, as shown in Fig. 1. Previously, patients on HFNC were observed in the POU for a maximum of 8 h before PICU transfer, whereas the new protocol permitted continued management in the POU. HFNC was initiated for patients who were persistently hypoxemic (SpO_2_ < 90%) and/or in respiratory distress, a clinical judgement made by a physician considering factors such as subcostal and intercostal retractions, nasal flaring, grunting, and tachypnea. Patients in the POU were transferred to a PICU at a nearby children’s hospital if they required escalation to positive pressure ventilation, as determined by oxygen requirement (SpO_2_ </= 92% on FiO_2_ >/= 50%) and continued respiratory distress. Measures to ensure safety around transfer included having a critical care–trained pediatric emergency medicine attending assist with patient management in the POU before transfer, as well as the presence of a critical care team during transfer. Staffing of respiratory therapists and nurses remained unchanged during protocol implementation. The new protocol was reviewed in on-unit sessions with registered nurses and during section meetings with attending physicians. Institutional review board approval was obtained for this study.

### Selection of participants

Patients who were clinically unstable, such as having altered mental status, or had significant co-morbidities, such as congenital heart disease or being immunosuppressed, were not eligible for POU admission. All other patients requiring respiratory support during the protocol implementation period were admitted to the POU. All patients admitted to the POU from October 2022 to January 2023 who required HFNC as a part of their care were included in the study.

### Measurements and outcomes

Demographic data, clinical history, hospital course, disposition, and clinical outcomes were collected during the chart review. Disposition from the POU included discharge home or transfer to the PICU. Respiratory support time was defined as the total amount of time spent on any form of respiratory support above low-flow oxygen (HFNC, CPAP, BiPAP, nasal synchronized intermittent mechanical ventilation [NSIMV]) during any part of the patient’s hospital stay (POU, PICU, and inpatient). Length of stay was defined as the total time spent in the hospital, from admission to POU to discharge home from POU or PICU/inpatient floor. PICU-bed-hours saved was calculated as the total sum of hours each patient received HFNC in the POU, including time up to the point of transfer for those requiring escalation to PICU.

### Data analysis

All data was entered into the institution’s REDCap (Nashville, TN). Descriptive statistics were tabulated in Microsoft Excel (Seattle, WA) and presented as median and interquartile range (IQR) for continuous variables and numbers and percentages for categorical variables.

## Results

From October 2022 to January 2023, 18 patients admitted to the POU requiring HFNC met criteria to be included in the study. Of these, 6 (33.3%) patients were discharged home from the POU and 12 (66.6%) were transferred to the PICU. Characteristics of these patients are listed in Table 1. Median age for the study population was 25.5 months (IQR 10.8–41.3). The median length of illness prior to presenting at the ED was 3.0 days (IQR 2.3-5.0). Most patients tested positive for RSV (10 [55.6%]), followed by influenza (5 [27.8%]). 8 (44.4%) patients in the sample had a final diagnosis of bronchiolitis only, 6 (33.3%) had pneumonia only, 3 (16.7%) had bronchiolitis with secondary pneumonia, and 1 (5.6%) was diagnosed with asthma exacerbation. 83.3% received albuterol, 61.1% received steroids, 11.1% received epinephrine, and 27.3% received magnesium while they were in the PED or POU (data not shown).

**Table 1:** Clinical characteristics of patients initiated on high-flow nasal cannula in the POU

	Total (*n* = 18)	Disposition from POU	
		Discharged home (*n* = 6)	Transferred to PICU (*n* = 12)
Age (months), median (IQR)	25.5 (10.8–41.3)	33.5 (13.8–66.8)	25.0 (10.8–38.3)
Weight (kg), median (IQR)	13.2 (10.4–15.8)	15.2 (10.7–22.0)	13.0 (10.8–14.1)
Female sex, n (%)	7 (38.9)	2 (33.3)	5 (41.7)
Days of illness prior to admission, median (IQR)	3.0 (2.3-5.0)	4.0 (3.0–5.0)	3.0 (2.0-4.8)
Signs or symptoms on admission, n (%)			
Fever	15 (83.3)	5 (83.3)	10 (83.3)
Shortness of breath	14 (77.8)	5 (83.3)	9 (75.0)
Wheezing	9 (50.0)	4 (66.7)	5 (41.7)
Pathogen of infection, n (%)			
RSV	10 (55.6)	3 (50.0)	7 (58.3)
Influenza	5 (27.8)	0	5 (41.7)
Other	3 (16.7)	3 (50.0)	0
Final diagnosis, n (%)			
Bronchiolitis only	8 (44.4)	2 (33.3)	6 (50.0)
Pneumonia only	6 (33.3)	3 (50.0)	3 (25.0)
Bronchiolitis + Pneumonia	3 (16.7)	1 (16.7)	2 (16.7)
Asthma exacerbation	1 (5.6)	0	1 (8.3)

*POU* pediatric observation unit, *PICU* pediatric intensive care unit, *IQR* interquartile range, *RSV* respiratory syncytial virus

Table 2 lists the clinical course of patients admitted to the POU. HFNC settings at initiation had a median flow rate of 1.44 L/kg/min (IQR 1.12–1.61) and FiO_2_ 40.0% (IQR 32.5–47.5), while the maximum HFNC settings over the hospital stay had a median flow rate of 1.83 L/kg/min (IQR 1.55–2.09) and FiO_2_ 40.0% (IQR 32.5–40.0). 4 of the patients transferred to the PICU required higher forms of oxygen support – one requiring CPAP, two requiring BiPAP, and one requiring NSIMV. 2 of these 4 patients required placement on positive pressure ventilation while in the POU before transfer. No patients in this cohort required intubation. Patients who were transferred to the PICU spent a median of 19.3 h (IQR 10.1–34.2) in the POU before the transfer. No adverse events occurred during transfer to the PICU.

**Table 2:** Clinical course of patients initiated on High-Flow nasal cannula in the POU

	Total (*n* = 18)	Disposition from POU			
		Discharged home (*n* = 6)			Transferred to PICU (*n* = 12)
Initial HFNC settings					
FiO_2_ (%)	40.0 (32.5–47.5)	40.0 (32.5–47.5)			40.0 (37.5–43.8)
Flow (L/kg/min)	1.44 (1.12–1.61)	1.22 (0.68–1.42)			1.52 (1.20–1.73)
Maximum HFNC settings					
FiO_2_ (%)	40.0 (32.5–40)	40.0 (32.5–40)			40.0 (37.5–42.5)
Flow (L/kg/min)	1.83 (1.55–2.09)	1.54 (0.77–1.70)			2.01 (1.79–2.15)
Time in POU (hours)	33.9 (16.5–48.8)	50.7 (38.4–59.9)			19.3 (10.1–34.2)
Respiratory support time (hours)	32.2 (16.5–58.0)	21.4 (14.1–33.2)			51.7 (21.3–63.3)
Length of stay (hours)	79.3 (60.2-101.5)	50.7 (38.4–59.9)			87.3 (79.6-125.4)

Data presented in median (IQR)

*IQR* interquartile range, *POU* pediatric observation unit, *PICU* pediatric intensive care unit, *HFNC* high-flow nasal cannula

Median length of stay of the whole cohort was 79.3 h (IQR 60.2-101.5). Among the 6 patients discharged home from the POU, median length of stay was 50.7 h (IQR 38.4–59.9) with a median of 21.4 h (IQR 14.1–33.2) on HFNC. Among the other 12 patients, transfer to PICU occurred a median of 19.3 h (IQR 10.1–34.2) into their POU course. In total, managing patients on HFNC in the POU resulted in 293.2 PICU-bed-hours saved. During the study period, POU admission volumes were 16.3% higher and POU length of stay 10.1% longer compared to the same period the previous year (data not shown). There were no deaths in our study sample, and no patients returned to the ED within 7 days of discharge.

## Discussion

In this study, we describe the use of HFNC in a community pediatric observation unit (POU) with no inpatient hospital unit on-site in a resource-limited setting due to a respiratory virus surge causing shortages in PICU beds. The data presented supports the use of observation units to safely manage children requiring HFNC therapy to improve utilization of intensive care beds during a resource-limited time period. There were no deaths and a zero-intubation rate among our sample of 18 patients, including 6 patients who were able to be discharged home after receiving HFNC in the POU.

Given the setting, it is not surprising that RSV and influenza were the most common pathogens in our sample. None of our patients tested positive for COVID-19. Incidentally, all patients who tested positive for influenza were transferred to the PICU. Those successfully discharged home from the POU included patients with both RSV and other respiratory viruses, such as rhinovirus. Those successfully managed on HFNC in the POU also had a wide range of ages, from 3 months old to 9 years old, and a variety of diagnoses, including bronchiolitis, pneumonia, and even more complicated disease courses such as bronchiolitis with secondary pneumonia. The one patient with an asthma exacerbation was eventually transferred to the PICU due to needing a higher level of respiratory support despite continuous albuterol. A recent review by Chao et al. provides mixed evidence on the use of HFNC in children with asthma, citing delays in initiating higher forms of respiratory support and difficulty delivering inhalation drugs [20]. Other studies have also shown more success in HFNC therapy in patients with bronchiolitis compared to other respiratory illnesses [21–23]. Nonetheless, our data suggests that patients with various disease processes can be initially managed in a POU safely especially in times where PICU resources are limited.

Median length of stay in the POU was around 34 h, slightly longer than described in previous studies, which averaged around or less than 24 h [15, 16]. This finding in our study could be due to the shortage of PICU beds during this period resulting in longer transfer wait times and so patients were held in our POU for longer than the usual 48-hour limit. On average, patients who needed higher level of care were identified and transferred within 24 h of being in the POU, and no patients in our sample returned to the ED within 7 days of discharge. Additionally, in this study, HFNC administration in the POU decreased PICU utilization by almost 300 bed-hours, supporting evidence from previous studies that managing pediatric respiratory illnesses in a POU setting can decrease hospital utilization and costs [17, 18]. This suggests the possibility of using observation units as safe clinical decision units to identify resource allocation, especially in conjunction with the growing literature on predictors of HFNC failure to identify appropriate candidates for POU management [24].

The viral respiratory surge did result in an increased work burden on POU providers as evidenced by the increased POU admission volume and length of the stay compared to the previous year. The population of patients requiring HFNC required more bedside care from nursing and respiratory teams, whose official staffing ratios did not change during the surge.

The biggest limitation of this study is the sample size, which was small at only 18 patients, limiting generalizability. Another limitation included some variations in clinical practice among attending providers in the POU despite implementation of a HFNC clinical pathway. This was particularly evident in the initiation of HFNC; in our sample, our median initiation flow rate was 1.44 L/kg/min, while the protocol recommends starting at 2 L/kg/min. This variation in clinical practice is likely attributable to individual provider preference. Furthermore, our protocol did not use a standardized respiratory scoring system, which also introduces variability in clinical judgement of respiratory distress. In the future, this could be improved upon by applying quality measures such as building the protocol into the electronic medical record, including a respiratory scoring system.

## Conclusion

HFNC has been traditionally limited to the PICU or inpatient floors. This study is the first known to suggest that it could be successfully used in an observation unit during a severe PICU bed shortage. This allows for prioritization of PICU resources for patients with the most severe disease manifestations. Further studies on the effectiveness of HFNC use in an observation unit setting are warranted, such as identifying the clinical characteristics or predictive models for children who can be safely cared for in the POU without subsequent PICU transfer. More detailed cost-benefit analyses are needed to fully explore the potential of using HFNC in an observation unit on improving healthcare resource utilization.

## Data Availability

The datasets used and/or analyzed during the current study are available from the corresponding author on reasonable request.

## Abbreviations

HFNC: high flow nasal cannula
CPAP: continuous positive airway pressure
BiPAP: bilevel positive airway pressure
FiO_2_: fraction of inspired oxygen
PICU: pediatric intensive care unit
RSV: respiratory syncytial virus
POU: pediatric observation unit
PED: pediatric emergency department
NSIMV: nasal synchronized intermittent mechanical ventilation
IQR: interquartile range
